# Exploiting genetic diversity in two European maize landraces for improving Gibberella ear rot resistance using genomic tools

**DOI:** 10.1007/s00122-020-03731-9

**Published:** 2020-12-03

**Authors:** David Sewordor Gaikpa, Bettina Kessel, Thomas Presterl, Milena Ouzunova, Ana L. Galiano-Carneiro, Manfred Mayer, Albrecht E. Melchinger, Chris-Carolin Schön, Thomas Miedaner

**Affiliations:** 1 State Plant Breeding Institute, University of Hohenheim, Stuttgart, Germany; 2 Kleinwanzlebener Saatzucht (KWS) KWS SAAT SE & Co. KGaA, Einbeck, Germany; 3 Institute of Plant Breeding, Population Genetics and Seed Science, University of Hohenheim, Stuttgart, Germany; 4 Plant Breeding, TUM School of Life Sciences Weihenstephan, Technical University of Munich, Freising, Germany

## Abstract

**Key message:**

High genetic variation in two European maize landraces can be harnessed to improve Gibberella ear rot resistance by integrated genomic tools.

**Abstract:**

*Fusarium graminearum* (Fg) causes Gibberella ear rot (GER) in maize leading to yield reduction and contamination of grains with several mycotoxins. This study aimed to elucidate the molecular basis of GER resistance among 500 doubled haploid lines derived from two European maize landraces, “Kemater Landmais Gelb” (KE) and “Petkuser Ferdinand Rot” (PE). The two landraces were analyzed individually using genome-wide association studies and genomic selection (GS). The lines were genotyped with a 600-k maize array and phenotyped for GER severity, days to silking, plant height, and seed-set in four environments using artificial infection with a highly aggressive Fg isolate. High genotypic variances and broad-sense heritabilities were found for all traits. Genotype-environment interaction was important throughout. The phenotypic (*r*) and genotypic (${r}_{g}$) correlations between GER severity and three agronomic traits were low (*r* =  − 0.27 to 0.20; ${r}_{g}\hspace{0.17em}$=  − 0.32 to 0.22). For GER severity, eight QTLs were detected in KE jointly explaining 34% of the genetic variance. In PE, no significant QTLs for GER severity were detected. No common QTLs were found between GER severity and the three agronomic traits. The mean prediction accuracies ($\rho$) of weighted GS (wRR-BLUP) were higher than $\rho$ of marker-assisted selection (MAS) and unweighted GS (RR-BLUP) for GER severity. Using KE as the training set and PE as the validation set resulted in very low $\rho$ that could be improved by using fixed marker effects in the GS model.

**Electronic supplementary material:**

The online version of this article (10.1007/s00122-020-03731-9) contains supplementary material, which is available to authorized users.

## Introduction

Ear rot infections caused by *Fusarium graminearum, F. verticillioides, Aspergillus flavus*, and/or *Stenocarpella maydis* are global threats to maize production. In Germany, a recent survey on the prevalence of *Fusarium* species showed that *F. graminearum* (Fg) and *F. verticillioides* (Fv) were dominant, their relative occurrence depending on temperature and humidity (Pfordt et al. 2020). *F. graminearum* (sexual stage: *Gibberella zeae*) causes Gibberella ear rot (GER) which reduces the quantity and quality of maize kernels and more importantly, contaminates the grains with mycotoxins such as deoxynivalenol (DON) and zearalenone (ZON) (Trail 2009; Ding et al. 2011; Martin et al. 2012a; Mesterházy et al. 2012). These mycotoxins are associated with serious health problems such as kidney diseases, poor growth, and disorders of reproduction in animals and humans (Pinton and Oswald 2014; Zhou et al. 2018). Empirical studies revealed high correlations between GER severity and DON as well as ZON contents in European maize by artificial infection with individual isolates (Bolduan et al. 2009; Martin et al. 2012a; Mesterházy et al. 2020). Because of the adverse health and economic effects of mycotoxins, regulatory bodies in most parts of the world have set recommended limits in maize kernels and products (FAO 2003; The Commission of the European Communities 2006; Foroud et al. 2019). An integrated disease management strategy can support existing efforts to reduce ear rots and associated mycotoxin contaminations in maize with GER resistant cultivars being an essential prerequisite.

In maize, GER resistance is inherited quantitatively with mostly small-effect quantitative trait loci (QTLs) (Xiang et al. 2010; Martin et al. 2012a). In the past years, a genome-wide association study (GWAS) was performed to identify QTLs for GER resistance using single-locus models (Han et al. 2018). However, multi-locus models such as fixed and random model circulating probability unification (FarmCPU, Liu et al. 2016) have been found to be more powerful in detecting SNP-trait associations with a lower rate of false positives and false negatives than single SNP-based models, especially for traits with complex genetic architecture (Abed and Belzile 2019; Kaler et al. 2020; Malik et al. 2019; Miao et al. 2019; Odilbekov et al. 2019; Wei et al. 2017; Xu et al. 2018; Zhang et al. 2019; Zhu et al. 2018).

For complex polygenic traits, genomic selection (GS) offers an attractive alternative to conventional or marker-assisted selection (Meuwissen et al. 2001). The potential of GS for improving quantitative resistances has been analyzed for several pathosystems, e.g., for resistance to lethal necrosis (Gowda et al. 2015), Diplodia ear rot (dos Santos et al. 2016) and Northern corn leaf blight (Technow et al. 2013). Two studies (Han et al. 2018; Riedelsheimer et al. 2013) investigated the prospects of GS for GER resistance in European elite maize lines.

Landraces serve as repositories of diverse alleles of agronomic importance and have great potential for broadening the genetic diversity of elite maize germplasm as illustrated for several agronomical traits (Yao et al. 2007; Strigens et al. 2013; Bedoya et al. 2017). European maize landraces have experienced several hundred years of adaptation to European growing conditions and can have a higher chance of successful allele transfer to elite backgrounds compared to non-adapted lines. The molecular diversity of 35 European maize landraces was investigated by Mayer et al. (2017) using high-density genotypic data and landraces, “Kemater Landmais Gelb” (KE, originating from Austria) and “Petkuser Ferdinand Rot” (PE, originating from Germany) represented a high proportion of the total molecular diversity (Mayer et al. 2017; Hölker et al. 2019). Thus, they were chosen for large-scale production of doubled haploid (DH) lines, which were extensively genotyped and phenotyped for numerous agronomic traits but not for Fusarium diseases (Hölker et al. 2019).

Our objective was to investigate the genetic architecture of Fg resistance in two DH libraries derived from landraces KE and PE and the potential of genetic improvement by marker-assisted (MAS) and genomic selection (GS). Specifically, we aimed to (1) estimate variances and covariances for GER severity and the agronomic traits, days to silking, plant height, and seed-set, (2) map QTLs using a multi-SNP GWAS model based on the markers from a 600 k SNP array, and (3) compare the prediction accuracies of MAS and two GS approaches for GER severity. Therefore, 250 DH lines from each landrace were artificially infected by *F. graminearum* in four environments.

## Materials and methods

### Plant materials, experimental design and data collected

Plant materials consisted of a panel of 500 DH lines produced from two European flint landrace populations, KE and PE by KWS SAAT SE & Co. KGaA, Einbeck, Germany. We phenotyped 250 DH lines per population plus 10 checks (including the two original source populations) in 2018 and 2019 at Hohenheim (HOH) near Stuttgart, Germany, and at Gondelsheim (GON) near Karlsruhe, Germany. The DH lines represent a random sample of the DH lines described by Hölker et al. (2019). The experimental design was a 51 × 10 alpha lattice design (10 genotypes per 51 incomplete blocks) with 2 replicates in both locations and years. Sowing was done mechanically. Each plot was a single row of 3 m length and consisted of 20 plants at intra-row spacing of 15 cm. Inter-row spacing was 75 cm. Eight to ten maize ears per plot, leaving out border plants, were inoculated with inoculum prepared from a highly aggressive *F. graminearum* (Fg) isolate, FG 163 (= IFA 66, Martin et al. 2012a, b) at a concentration of 1.5 × 10^4^ spores mL^−1^. The isolate was shared by Prof. Marc Lemmens, BOKU, Vienna, Austria. Each upper ear was inoculated by a one-needle vaccinator on the silk channel of the maize cobs with approximately 2 ml of the inoculum at 4–6 days after 50% silk emergence (Reid et al. 1996). Though significant genotype-isolate interaction for ear rot severity and DON content was reported in previous studies, Miedaner et al. (2010) found no rank reversals for GER resistance in early maize inbred lines inoculated with eight *F. graminearum* isolates where our isolate used in this study was one of them. Therefore, inoculation with one highly aggressive isolate should be adequate to discriminate resistant and susceptible lines. Days to silking (DS), plant height (PHT, cm), seed-set (SS, %), and GER severity (%) were recorded in all 4 environments (= location × year combinations). Briefly, days to silking were recorded as the number of days taken to achieve ≥ 50% female flowering per plot. PHT was measured plotwise from ground level to the first tassel branch using a meter rule in cm. At physiological maturity (about 18–20% grain moisture), we manually dehusked each ear and assessed visually seed-set as the proportion of kernels per cob (%), where 0% = no kernels on the cob and 100% = cob fully covered with kernels. GER severity was visually assessed on the same ears on a quantitative scale from 0 to 100%, where 0% = no Fg mold visible and 100% = entire ear covered with Fg mold.

## Data analysis

### Phenotypic analysis

ASReml R package version 3.0 (Butler 2009) was used to estimate means and variance components for all four traits. Trait values from each environment were used to calculate best linear unbiased estimates (BLUEs), regarding genotypes as fixed effects. Estimates of variance components and best linear unbiased predictors (BLUPs) were calculated by the following model, regarding genotypes within each population and the other factors as random:

$$ Y_{ijklm} = \mu + P_{i} + G_{j\left( i \right)} + E_{k} + R_{l\left( k \right)} + B_{{m\left( {kl} \right)}} + PE_{ik} + GE_{jk} + e_{ijklm} $$

where ${Y}_{ijklm}$ = the observed phenotypic value for genotype *j* from population *i* in replicate *l* and block *m* at environment *k*, $\mu$ = general mean, ${P}_{i}$ = effect of the *i*th population, ${G}_{j(i)}$ = effect of the *j*th genotype nested in the *i*th population, ${E}_{k}$ = effect of the *k*th environment, ${R}_{l(k)}$ = effect of the *l*th replicate nested in the *k*th environment, ${B}_{m(kl)}$ = effect of the *m*th block nested in the *l*th replicate and the *k*th environment, $P{E}_{ik}$ = interaction effect between the *i*th population and the *k*th environment, ${GE}_{Jk}$ = interaction effect between the *j*th genotype and the *k*th environment, and ${e}_{ijklm}$ = residual error. We assumed heterogeneous variances of residual effects in different environments. Dummy variables were used to separate the genotypes into checks and the two landraces (KE and PE) in the random statement to obtain the variance components for each population (Piepho et al. 2006). The likelihood ratio test based on full and reduced models was used to determine the significance of variance components. The same model was used for calculations in individual environments by omitting the environment factor. Repeatabilities and broad-sense heritabilities (H^2^) were estimated by standard procedures described by Hallauer et al. (1988). Pearson correlation coefficient (*r*) between BLUEs of traits were estimated using the function “cor.test” in R programming language (R Core Team 2018). Genotypic correlations (${r}_{g}$) between traits and their *P*-values were calculated using bivariate models described in details by Wilson et al. (2010), in Asreml-R 3.0 (Butler 2009).

## Molecular analysis

The 500 DH lines (250 derived from PE and KE, respectively) were previously genotyped using a high-density Affymetrix® Axiom® Maize Genotyping Array optimized for temperate maize (Unterseer et al. 2014, Mayer et al. 2020). SNP markers having call rate < 90%, minor allele frequency < 5%, and too high heterozygosity (false discovery rate < 1%) were excluded from the marker data. The remaining heterozygous loci were replaced with missing values, and the new data set without heterozygous loci was filtered again as described above. Remaining missing values were imputed using Beagle 5.0 software (Browning et al. 2018). A total of 388,999 SNPs and 462 DH lines (KE = 236, PE = 226) were left for further statistical analyses after quality check. Physical positions of all markers are available on the public maize reference genome, B73 RefGen_v4, AGPv4 (Jiao et al. 2017).

### Principal component analysis and genomic kinship

Principal component analysis (PCA) was carried out by the default method in the R package Genome Association and Integrated Prediction Tool (GAPIT) 3.0 (Lipka et al. 2012). In addition, a kinship plot was created from the genomic relationship matrix of the high-density SNP marker data using the default kinship.algorithm, VanRaden (VanRaden 2008) in GAPIT 3.0 (Lipka et al. 2012).

### Genome-wide association studies (GWAS)

The BLUEs and the high density filtered SNPs were used to perform GWAS for GER severity (%), DS (days), PHT (cm), and SS (%), employing the multi-locus-based method, FarmCPU (Liu et al. 2016) implemented in the R package GAPIT 3.0 (Lipka et al. 2012). The GWAS was conducted with the filtered DH lines from each population (KE = 236 and PE = 226) separately. In FarmCPU, false positives are controlled by using a special kinship (K) matrix created from pseudo-quantitative traits nucleotides (pseudo-QTNs) as random effect (Liu et al. 2016). The parameter, “method.bin” was set to “optimum” for the optimization process, using the default bin.size = c(5e5,5e6,5e7) and bin.selection = seq(10,100,10). The “bin.size” function refers to the division of the whole genome into bins in kilo base pairs and represents the window size used to select a probable QTN. The “bin.selection” indicates the number of possible QTNs that can be selected into the model as covariates in loops. After the optimization process in a random effect model, the marker having the most significant *P*-value in a particular bin is used to represent that bin (Liu et al. 2016). The two steps of FarmCPU model, which are run iteratively are described in detail by Liu et al. (2016) and can be represented as.

- Step 1. Fixed effect model (FEM): ${y}_{j}={M}_{j1}{T}_{1}+{M}_{j2}{T}_{2}+...+{M}_{jt}{T}_{t}+{S}_{jn}{e}_{n}+{\varepsilon }_{j}$
- Step 2. Random Effect Model (REM): ${y}_{j}={u}_{j}+{\varepsilon }_{j}$

In both FEM and REM, ${y}_{j}$=the trait value (i.e., BLUE across environments) of the *j*th maize DH line and ${\varepsilon }_{j}$= residual ~ *N*(0,${\sigma }_{\varepsilon }^{2}$). In FEM, ${M}_{j1}, {M}_{j2}, ..., {M}_{jt}$= the genotypes of *t* pseudo-QTNs, initiated as an empty set (Liu et al. 2016), ${T}_{1}, {T}_{2}, ..., {T}_{t}$= the corresponding effects of the pseudo-QTNs; ${S}_{jn}$ = the genotype score of the *j*th DH line at the *n*th SNP marker and ${e}_{n}$ = the corresponding effect of the *n*th SNP marker. In REM, ${u}_{j}$= the total genetic effect of the *j*th DH line, where the variance and covariance matrix is represented by $G=2K{\sigma }_{g}^{2}$, $K$= the kinship matrix constructed based on the pseudo-QTNs and ${\sigma }_{g}^{2}$ = the genetic variance pertaining to the REM (Liu et al. 2016).

In order to identify which SNPs were most likely associated with each trait, we adopted an exploratory significant threshold of *P*-value ≤ 0.0001 (–log10 (*P*-value) ≤ 4) and Bonferroni-corrected threshold of (–log10 (*P*-value) = 6.89). The total proportion of genotypic variance (${p}_{G}$) explained by the QTLs detected were calculated using the formula.

$$ {\text{p}}_{{\text{G}}} = \frac{{R_{{{\text{adj}}}}^{2} }}{{H^{2} }} $$

where ${H}^{2}$ is the broad-sense heritability of the trait, and ${R}_{adj}^{2}$ is the adjusted ${R}^{2}$ from a linear model (Utz et al. 2000). Calculation of ${R}_{adj}^{2}$ and ${p}_{G}$ for (a) a simultaneous fit of all significant QTL and (b) individual QTL followed the procedure described by Würschum et al. (2015).

## Candidate gene identification for GER resistance

We searched for possible genes for GER resistance using the publicly available B73 reference genome version 4 (Zm-B73-REFERENCE-GRAMENE-4.0, Jiao et al. 2017) from MaizeGDB (https://www.maizegdb.org/gene_center/gene) based on the positions of two most important SNPs explaining > 5% of genotypic variance for GER resistance in KE (i.e., ZmSYNBREED_24070_673 on chromosome (chr.) 2 and ZmSYNBREED_53695_527 on chr. 6). Descriptions and ontology terms of genes located within ≤ 1 cM (approx. ≤ 250 kb) around the SNPs (Coan et al. 2018) were obtained from the Gramene Annotations (http://www.gramene.org/).

## Marker-assisted and genomic selection for GER severity

We evaluated the potential of GS for GER resistance using two models, ridge regression-BLUP (RR-BLUP) and weighted ridge regression-BLUP (wRR-BLUP) using the R package “rrBLUP” (Endelman 2011; Endelman and Jannink 2012). In wRR-BLUP, the significant SNPs from the GWAS explaining > 5% ${p}_{G}$ for GER severity were fitted in the GS model as a fixed effect, and all other SNPs fitted as a random effect (Zhao et al. 2014; Spindel et al. 2016). In addition, we compared the prediction accuracies of marker-assisted selection (MAS, i.e., by using the significant SNPs from the GWAS explaining > 5% ${p}_{G}$) and the two genome-wide prediction models (RR-BLUP and wRR-BLUP) for GER resistance.

The quality of prediction of these models was evaluated by cross-validation using 80% of the data as training set (TS) and the remaining 20% as validation set (VS) (Liu et al. 2013; Würschum et al. 2014; Würschum and Kraft 2014). Sampling was stratified by landrace population and repeated 1000 times. To reduce computation time, we did not perform a de novo QTL detection for each calibration set for the MAS and wRR-BLUP. Instead, we predicted based on QTL positions and effects detected in the whole dataset. For RR-BLUP and wRR-BLUP, we also investigated the prediction accuracy of GS for GER resistance across the two landraces. Here, KE was exclusively used as the TS and PE as the VS, and vice versa. The prediction accuracy ($\rho$) was determined by expressing the predictive ability (i.e., correlation coefficient between the observed BLUEs and the predicted values) as a fraction of the square root of the broad-sense heritability of the trait. The model used to estimate marker effects in the TS is given by the following:

$$Y=X\beta +Zu+e$$

where = $Y$ the vector of BLUEs for GER; $\beta$ = vector of fixed effects; $u \sim N(0,A\sigma_{u}^{2} )$ = the vector of random marker effects, $A$ is a relationship matrix and the residuals are normal with constant variance; $X$ and $Z$ = the design matrices; $e$ = the residual error (Endelman 2011). We calculated the genomic estimated breeding values (GEBV) of the individuals of the VS by using the relation.

$${Y}_{0}={X}_{0}\beta + {Z}_{0}u$$

where ${Y}_{0}$= the vector of GEBV of the VS; ${X}_{0}$ and ${Z}_{0}$ = design matrixes of individuals in the VS. The predictions were based on additive effects of markers.

## Results

### Phenotypic and genetic variation for GER resistance and agronomic traits

**Fig. 1:**
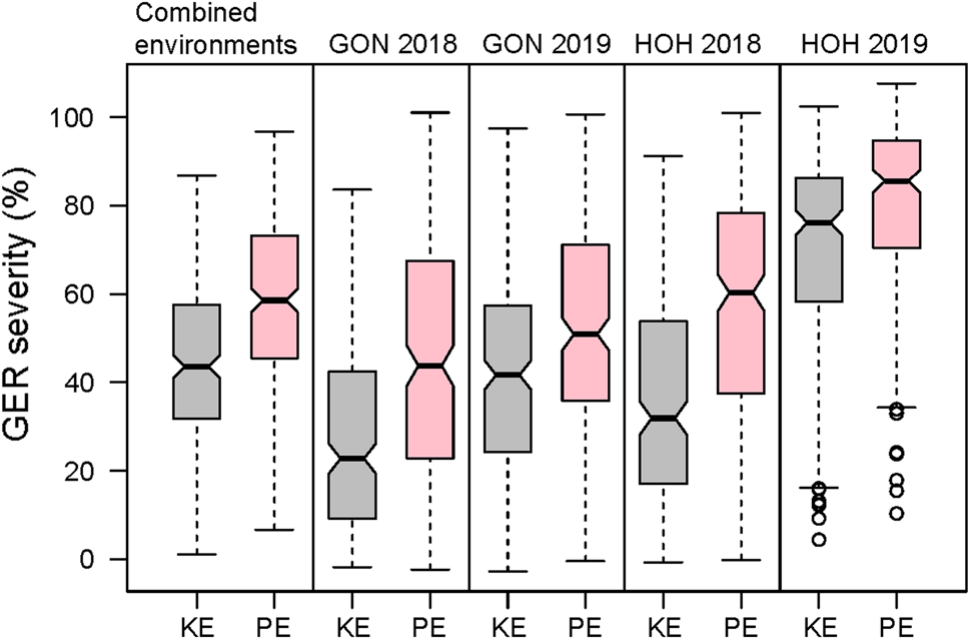
Box plots of adjusted means for Gibberella ear rot (GER) infection among Kemater (KE) and Petkuser (PE) DH lines at Gondelsheim (GON) and Hohenheim (HOH) in 2018 and 2019 plus the four environments combined. Horizontal thick lines in boxes indicate the median

**Fig. 2:**
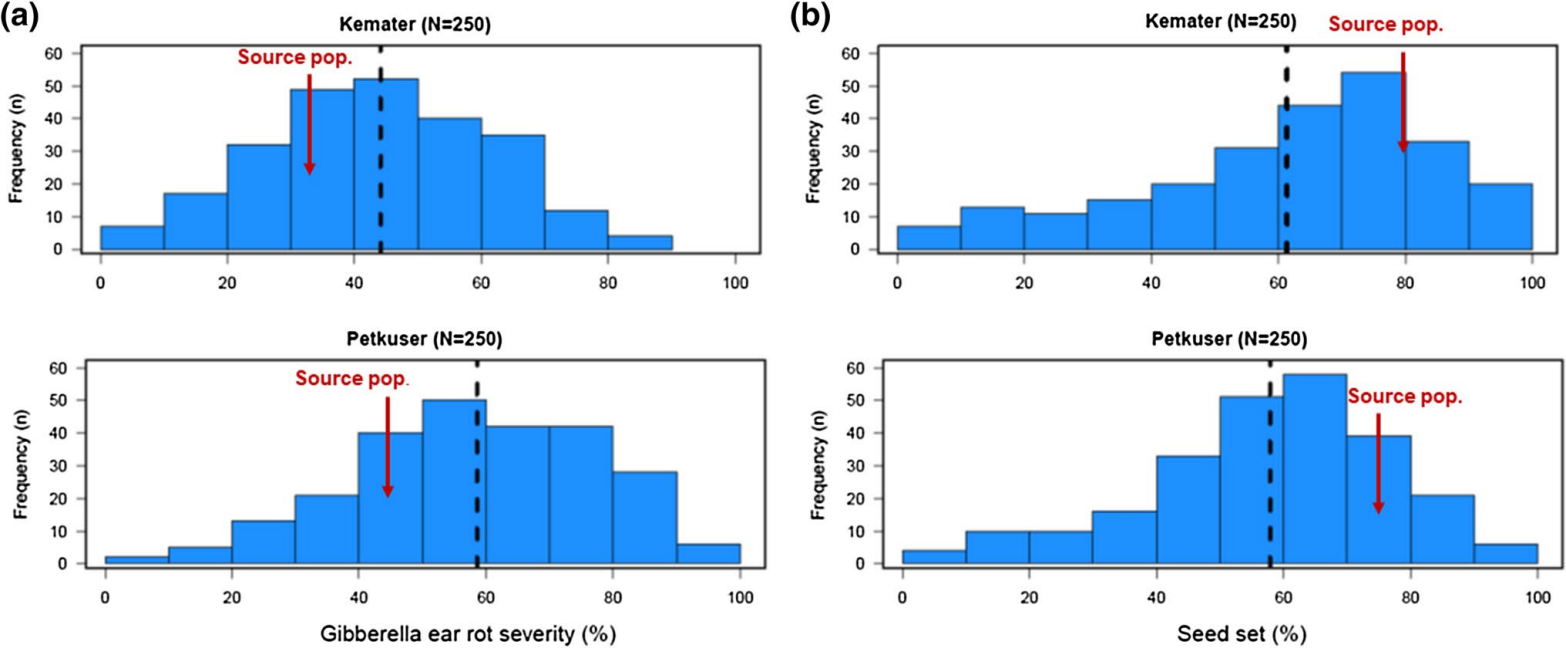
Histograms showing the distribution of **a** Gibberella ear rot** (**GER) severity and **b** seed-set among 250 DH lines within each landrace, across four environments. The red arrows indicate the mean value of GER severity and seed-set for the respective source populations (replicated 4-fold). Vertical dashed lines represent the mean disease severity and seed-set of DH lines

GER symptoms were observed among maize lines in all four environments with the highest mean severity in HOH 2019 and the lowest in GON 2018 (Fig. 1). Repeatability values per environment were moderate to high, ranging from 0.61 to 0.96, depending on the trait (Supplementary Table 1). Across the four environments, KE source population was slightly more resistant than PE source population (Fig. 2a). Accordingly, KE DH lines had a lower mean GER severity than PE lines (Fig. 1 and Fig. 2a). Variation within each population was high, GER severity ranging from 1 to 87% for KE and 7% to 97% for PE. On average, KE lines were about 25 cm taller than PE lines while DS was similar for both landraces. The average SS was slightly higher for KE than PE lines. Accordingly, the KE source population had a slightly higher seed set than the PE population (80% *vs*. 75%) (Table 1, Fig. 2b). We found significant (*P* ≤ 0.0001) genotypic and genotype–environment interaction variances and high H^2^ estimates for all traits (Table 1). H^2^ was higher for KE than PE for most traits. Phenotypic and genotypic correlations between GER severity and DS and SS were significant (*P* ≤ 0.01) in most cases but very low and similar for KE and PE (Table 2). DS was significantly and moderately correlated with SS. No significant correlations were found between GER severity and plant height.

**Table 1:** Means, genotypic variance (${\sigma }_{G}^{2}$), genotype-environment interaction variance (${\sigma }_{G\times E}^{2}$) and residual variance (${\sigma }_{\varepsilon }^{2}$) components and broad-sense heritabilities (${H}^{2}$) of Gibberella ear rot (GER) severity, days to silking (DS), plant height (PHT), and seed-set (SS) within landraces

Parameter	GER (%)	DS (days)	PHT (cm)	SS (%)
*Kemater (KE)*				
Mean	44.12	80.44	133.61	61.34
${\sigma }_{G}^{2}$	251.30	14.65	377.49	473.83
${\sigma }_{G\times E}^{2}$	94.64	2.00	53.35	123.45
${\sigma }_{\varepsilon }^{2}$	305.01	2.53	85.10	182.04
${H}^{2}$	0.80	0.95	0.94	0.90
*Petkuser (PE)*				
Mean	58.57	79.86	108.70	57.88
${\sigma }_{G}^{2}$	255.60	14.75	324.03	302.01
${\sigma }_{G\times E}^{2}$	146.95	3.57	44.57	143.16
${\sigma }_{\varepsilon }^{2}$	305.01	2.53	85.10	182.04
${H}^{2}$	0.77	0.92	0.94	0.84

${\sigma_{G}^{2}}$ and $\sigma_{G \times E}^{2}$ for all traits and populations were significantly different from zero at *P* < 0.0001

**Table 2:** Phenotypic and genotypic (in brackets) correlations between Gibberella ear rot (GER) severity and days to silking (DS), plant height (PHT), and seed-set (SS) within landraces across four environments

Traits	DS	PHT	SS
*Kemater DH*			
GER severity	− 0.25 ( − 0.32)***	− 0.03 ( − 0.04)	0.20 (0.22)**
DS			− 0.52 ( − 0.56)***
*Petkuser DH*			
GER severity	− 0.27 ( − 0.31)***	− 0.05 ( − 0.06)	0.18 (0.22)**
DS			− 0.54 ( − 0.59)***

**,***Significantly different from zero at *P* ≤ 0.01 and *P* ≤ 0.0001, respectively (for both the phenotypic and genotypic correlations)

## Principal component analysis and genomic relationship

The PCA grouped the 462 DH lines used for the molecular analyses into two major clusters corresponding to the two maize landrace populations, KE and PE (Supplementary Fig. 1). The first, second, and third PCs explained 16.75%, 3.36%, and 3.25% of the molecular variation, respectively. Within KE, the percentage of variation explained by the first three PCs were 7.27%, 4.41%, and 4.16%, respectively. Similarly, among PE lines, the first three PCs explained 8.56%, 5.22%, and 3.47% of the molecular variation, respectively. The genomic relationship plot also showed two major groups corresponding to KE and PE landraces, with smaller sub-clusters within each landrace (Supplementary Fig. 2).

## QTLs for GER severity

**Fig. 3:**
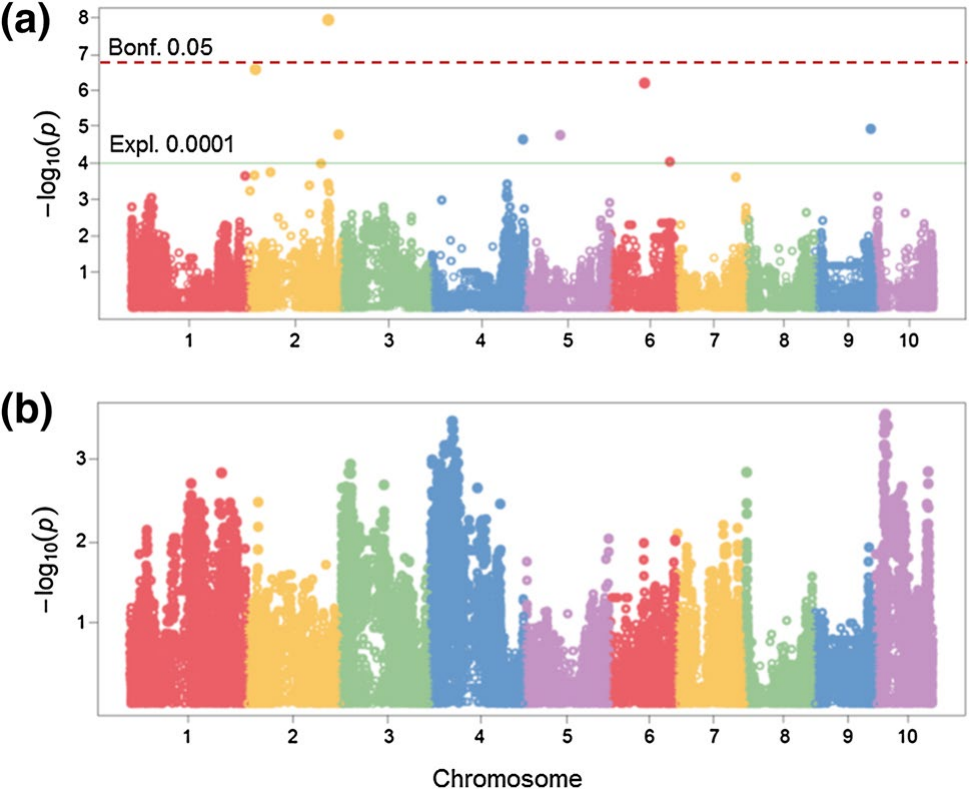
Manhattan plot of the GWAS scan for Gibberella ear rot (GER) severity in **a** “Kemater Landmais gelb” (*N*=236), and **b** ”Petkuser Ferdinand rot” (*N*=226). ***Ex*** ***pl.*** Exploratory threshold at *P* ≤ 0.0001; ***Bonf. ***Bonferroni-corrected threshold at *P* ≤ 0.05

Among KE DH lines (*N* = 236), at *P* = 0.0001, 8 QTLs collectively explaining 34% of ${p}_{G}$ for GER severity were found (Fig. 3a, Table 3). One SNP on chr. 2 (ZmSYNBREED_29737_831) exceeded the Bonferroni threshold (Fig. 3, Table 3). We detected 13, 11, and 1 QTL(s) for DS, PHT, and SS, respectively. None of the QTLs identified for GER severity colocalized with the QTLs detected for the agronomic traits in KE (Supplementary Table 2).

**Table 3:** Significant SNPs detected for Gibberella ear rot (GER) severity, their chromosomal position, *P*-value, frequency of the favorable allele (FAF), additive effects and proportion of explained genotypic variance (${p}_{G}$) in “Kemater Landmais gelb” (KE)

Marker	Chr^a^	Coordinate (cM)	*P*-value	FAF	Additive effect	${p}_{G}$ (%)
ZmSYNBREED_24070_673	2	49.00	2.70E–07	0.42	5.00	15.04
ZmSYNBREED_29737_831	2	119.54	1.17E–08	0.26	4.56	1.28
ZmSYNBREED_30537_486	2	162.00	1.70E–05	0.41	− 3.33	2.84
ZmSYNBREED_44869_210	4	162.93	2.33E–05	0.36	3.27	4.35
ZmSYNBREED_47633_944	5	78.30	1.75E–05	0.47	3.41	3.27
ZmSYNBREED_53695_527	6	31.15	6.36E–07	0.50	− 3.52	6.04
ZmSYNBREED_55609_889	6	91.78	9.50E–05	0.67	− 3.14	0.46
ZmSYNBREED_70955_321	9	110.30	1.18E–05	0.19	− 4.11	3.53
Total						33.69

^a^Chromosome

For PE (*N* = 226), no QTL were detected for GER severity at *P* = 0.0001 (Fig. 3b). SNP-GER resistance associations among PE lines were found at or near some of the loci identified in KE only with a lower significance level (e.g., *P* ≤ 0.01). Ten QTLs were detected for DS and PHT while one QTL was detected for SS in PE (*P* = 0.0001, Supplementary Table 3).

The two most important SNPs with the largest ${p}_{G}$ for GER severity in KE (i.e., ZmSYNBREED_24070_673 and ZmSYNBREED_53695_527) were placed in 25 protein-coding genes/gene models in the chosen interval, which could be placed into 10 functional categories (Supplementary Table 4).

## Genomic prediction *versus* marker-assisted selection for GER resistance

**Fig. 4:**
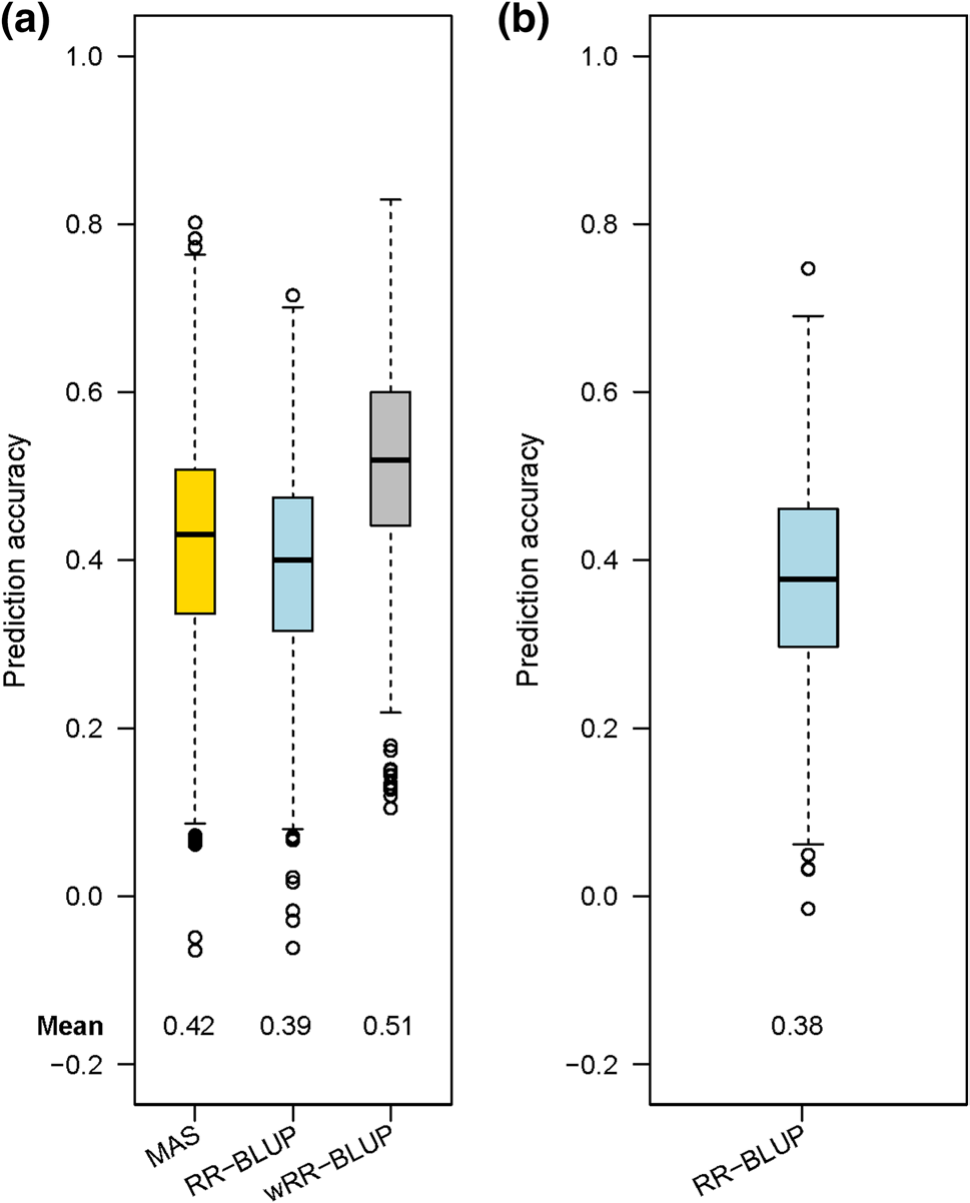
Box plots showing the prediction accuracies (%) of marker-assisted selection (MAS), ridge regression-BLUP (RR-BLUP) and weighted RR-BLUP (wRR-BLUP) models for Gibberella ear rot severity (%) in **a** “Kemater Landmais Gelb”, **b** “Petkuser Ferdinand Rot”

We evaluated the prospects of MAS and GS for GER resistance. For KE, we used the two SNPs explaining > 5% ${p}_{G}$ from the GWAS for MAS (Zhang et al. 2005) and all 388,999 markers for GS by adopting two models, RR-BLUP and wRR-BLUP. In wRR-BLUP, we used the medium-to-large-effect SNPs associated with GER QTLs as fixed effects as described in the Material and Methods. For KE (N_TS_ = 189, N_VS_ = 47), MAS and RR-BLUP yielded similar $\rho$ for GER severity (~ 0.40) while wRR-BLUP yielded the highest $\rho$ (0.51, Fig. 4). In PE (N_VS_ = 181, N_VS_ = 45), the mean $\rho$ of RR-BLUP was 0.38 (Fig. 4b). For GS across the two landraces, when only DH lines from KE were used as TS and PE as VS in RR-BLUP, $\rho$ for GER severity was 0.03 and vice versa $\rho$ was -0.01. When the two SNPs explaining > 5% ${p}_{G}$ for GER severity in KE were used as fixed effects in the GS model (wRR-BLUP), $\rho$ increased to 0.22 when KE lines constituted the TS and PE lines the VS.

## Discussion

Over the past decade, only few phenotypic and molecular studies have been conducted on GER resistance in elite maize germplasm compared to wheat (Gaikpa and Miedaner 2019) and exploitation of the genetic diversity among European flint maize landraces for GER resistance using genomic tools has not been pursued hitherto. In this study, we conducted GWAS and GS for GER resistance in two European flint maize landraces (KE from Austria and PE from Germany). To analyze potential covariations, we additionally evaluated days to silking (DS), plant height (PHT), and seed-set (SS).

## Variation for GER severity and agronomic traits in European landraces

Inoculation with the highly aggressive *F. graminearum* isolate FG163 resulted in GER infection in all locations and years. The environment and its interaction with the DH lines influenced GER severity in both landraces. Although the genotypic variances were quite similar for both landraces, PE showed higher genotype × environment variance resulting in a slightly higher H^2^ for KE (Table 1). A large effect of the environmental conditions on ear rot resistance has been reported several times in the literature (Giomi et al. 2016; Han et al. 2018; Galić et al. 2019; Morales et al. 2019). Therefore, it is important to phenotype lines for GER resistance in multi-environmental trials. The broad-sense heritability values were similar to previous reports (Martin et al. 2012a, b; Giomi et al. 2016; Han et al. 2016; Kebede et al. 2016).

We analyzed additionally agronomic traits such as DS, PHT, and SS, because they may lead to physiological escape or could have pleiotropic effects on GER resistance. All three traits had high H^2^ estimates ranging from 0.84 to 0.95 (Table 1). Within each population, low correlations (*r* =  − 0.27 to 0.20; ${r}_{g}$ =  − 0.32 to 0.22) were found between GER severity and the three agronomic traits analyzed, though significant in most instances (Table 2). Similarly to our findings, Han et al. (2018) and Martin et al. (2012c) reported low negative correlations between GER severity and days to silking illustrating that late materials tend to get less infected.

In wheat, PHT can highly affect severity of Fusarium head blight infection (Mesterházy 1995; Gaikpa et al. 2020), but this was not the case in our study with maize as judged from the non-significant correlations between PHT and GER severity (Table 2). This might be explained by the direct inoculation of the maize ears by hand while in wheat spray inoculation from above is commonly practiced.

Seed-set is an important fertility and yield-related trait in maize and also highly affected by inbreeding depression. DH lines from landraces are known to suffer more from inbreeding depression (Böhm et al. 2017; Strigens et al. 2013) because they have not experienced several cycles of inbreeding like elite material. *Fusarium* species are notorious in benefitting from host stress and the proportion of kernels on maize cobs itself might influence GER severity because many missing kernels reduce the nutrient ability of the fungus. In both cases, a close correlation between GER severity and SS should occur. The respective correlation coefficients between the two traits were significant, but low (Table 2). This implies that those DH lines that were heavily affected by inbreeding depression and consequently showing a low SS did not systematically suffer more from GER. The large differences in SS (Fig. 2b) within our populations might have been caused also by variation in flowering date as indicated by moderate negative correlations between both traits (*r* =  − 0.52 to  − 0.54, ${r}_{g}$ =  − 0.59 to  − 0.56, *P* < 0.0001). Genotypes flowering late had reduced seed-set, but this might have been caused by unusually low rainfall and higher temperatures towards the end of the silking period in 2018 and partially also in 2019 rather than by inbreeding depression. PHT is also affected by inbreeding depression, but the association between PHT and SS was not significant (${r}_{g}$ = 0.02 in KE and  − 0.06 in PE). Hence, we conclude that in our study GER was not strongly affected by inbreeding depression among lines. This is supported by the fact that the mean of the DH lines is not drastically higher than the mean of the source populations (Fig. 2a).

Our findings corroborate earlier studies reporting a high amount of genetic variation among landraces for Fusarium ear rot caused by *F. verticillioides* (Böhm et al. 2017). In the latter study, some DH lines from landraces were even less susceptible to FER than elite maize lines. The high phenotypic variation observed in this study can be exploited for GER resistance breeding and can be used for genomic-based approaches, like GWAS and GS. GER resistance was not much affected by the three agronomic traits and can, thus, be selected without undesirable correlated response.

## Marker-trait associations for GER severity among maize landraces

Although genotypic variation for GER severity found within PE was similar to KE (Table 1), no significant QTLs could be detected in PE, whereas in KE, we detected eight QTLs. This is astonishing as both landraces were evaluated in the same environments, with about the same number of genotypes (*N* ~ 230) and a high-density marker array (*N* = 388,999). As the complexity of a trait highly affects the outcome of the molecular analyses (Schön et al. 2004), many small-effect QTLs with rare alleles might control GER resistance in PE that could not be detected by GWAS at the chosen significant threshold. Though we did not find QTLs for GER severity within PE, QTLs were detected for the three other agronomic traits evaluated (*P* = 0.0001, Supplementary Table 3). In GWAS, strong linkage disequilibrium (LD) between a marker and a QTL allele is required to detect minor-effect QTLs (van Inghelandt et al. 2011). As LD decay was faster in PE than in KE (Mayer et al. 2017, 2020), this might partly explain the difference in QTL detection between both populations. Additionally, the presence of rare alleles in a population can result in low QTL detection power (Korte and Farlow 2013). At most of the QTL positions, minor alleles improved GER resistance in KE (Table 3). Also, the study of Han et al. (2018) found no QTLs for GER severity by GWAS in a line sortiment, but several QTLs for DON content, some of which were located in the same bin (2.02) where GER QTLs were identified in this present study (Table 3). Similar to the present outcome, QTLs have been reported on chromosome bins 2.03, 5.04, 6.07, and 9.05 for GER resistance in previous linkage mapping studies (Giomi et al. 2016; Han et al. 2016; Martin et al. 2011, 2012b). Although we found several QTLs for DS, PHT, and SS, none colocalized with QTLs for GER severity in KE. This accords to the low *r* and *r*_*g*_ found between GER and the agronomic traits.

Our molecular results agree with the assumption that GER resistance is controlled by many loci each contributing a small effect to the total genetic variation. Most intermediate and small-effect QTLs remain undetected in QTL mapping with small population size and lead to overestimation of the genotypic variance explained by the few detected QTLs (Beavis 1998; Melchinger et al. 1998; Schön et al. 2004; Xu 2003). Thus, larger population sizes are required to obtain an unbiased estimate of the proportion of explained genotypic variance of detected QTLs. The unexplained genetic variance by the QTLs detected in KE might be explained by QTLs with small additive effects that were below the significant threshold and QTLs with non-additive genetic effects on GER severity. Increase in population size and precision of disease ratings as well as exploration of GWAS models that can account for non-additive QTL effects are recommended. In an analysis combining both landraces KE and PE, however, we could not detect more QTL than in KE alone when including population (KE and PE) as a fixed effect in the model.

## Candidate genes associated with GER resistance

The two prioritized SNPs, ZmSYNBREED_24070_673 (chr. 2), and ZmSYNBREED_53695_527 (chr. 6) detected for GER resistance in KE, were associated with candidate genes which code for proteins belonging to families like cytochrome P450, mitogen-activated protein kinase kinase kinase (MAP3Ks), serine/threonine kinase, tetratricopeptide repeat (TPR)-like superfamily protein, leucine-rich repeat (LRR) family protein and armadillo (ARM) repeat superfamily protein. They are associated with functional groups such as binding activities, kinase activity, response to stress/stimulation, signal transduction, catalytic activity, metabolic and biosynthetic processes (Supplementary Table 4). Similar functional categories were reported for differentially expressed genes for *F. graminearum* (Yuan et al. 2020) and *F. verticillioides* (Fv, Yao et al. 2020) resistances in maize.

In previous studies, cytochrome P450 metabolism was found to be involved in Fv resistance in maize (Yao et al. 2020) because it regulates lipid metabolism and influences the production and activity of jasmonic acid as well as synthesis of secondary metabolites such as flavonoid and plant hormones (Koo et al. 2011). Mitogen-activated protein kinases (MAPKs) are highly conserved and transduce signals from the environment into cellular response in plants (Sopeña-Torres et al. 2018). MAP3Ks YODA found in the present study was previously reported to confer broad-spectrum resistance to fungi, bacteria, and oomycetes in *Arabidopsis* (Sopeña-Torres et al. 2018). Additionally, a combined linkage mapping or GWAS and transcriptomic data identified kinase genes for Fv resistance in maize (Maschietto et al. 2017; Yao et al. 2020). Han et al. (2018) also found a protein serine/threonine kinase annotated gene on chr. 2 associated with DON accumulation in maize. The significant roles of TPR-like superfamily protein, LRR family protein and ARM repeats in biotic and abiotic stress regulations have been extensively documented (Shanmugam 2005; Rosado et al. 2006; Padmanabhan et al. 2009; Sharma and Pandey 2016) and LLR family protein has been validated to control *A. flavus* resistance in maize (Dhakal et al. 2017).

## Weighted genomic selection outperformed marker-assisted selection for GER resistance

In practice, independent TS and VS are used for GS. However, in this study, we simulated the prospect of MAS and GS in the same experimental material using a fivefold cross-validation procedure (Liu et al. 2013; Würschum et al. 2014; Würschum and Kraft 2014). Additionally, the prospect of using each landrace population exclusively as TS or VS was evaluated. Within KE, the average prediction accuracy of MAS and unweighted GS (RR-BLUP) were similar implying that the QTLs detected by the multi-locus GWAS model (FarmCPU) were able to capture most of the important additive variance controlling GER severity. MAS is expected to yield better predictions only when major QTLs are underlying a trait, *e.g., Fhb1*, *Fhb2, Fhb4*, *Fhb5* for Fusarium head blight resistance in wheat (Buerstmayr et al. 2002; Ma et al. 2020). The $\rho$ estimated by RR-BLUP was similar for both, KE and PE DH libraries (39% and 38%, respectively). In RR-BLUP, the effects of many QTLs with small effects are estimated simultaneously and can result in underestimation of the effects of major genes in a population (Bernardo 2014). In contrast, the weighted GS (wRR-BLUP) approach outperformed MAS and RR-BLUP (Fig. 4). Therefore, we hypothesize that different information is captured by the fixed compared to the random effects (Spindel et al. 2016). The superiority of wRR-BLUP agrees with the findings for Fusarium head blight and Septoria tritici blotch resistance in small-grain cereals (Galiano-Carneiro et al. 2019; Herter et al. 2019; Odilbekov et al. 2019). However, estimates of MAS and wRR-BLUP are likely to be somewhat inflated in our study, because we based predictions in the VS on QTLs detected from GWAS in the entire data set. An alternative for getting an unbiased estimate would be the cross-validation procedure suggested by Utz et al. (2000). However, this procedure would be computationally very demanding for our study with about 390,000 markers as it requires in each of the *n* runs (1) performing GWAS for QTL detection and (2) establishing the GS model with 80% of the population in the training set, and application of the model to the remaining 20% of the population. The unweighted GS approach is a possibility when most of the low-effect QTLs underlying a trait cannot be detected by a GWAS model like in PE.

A close relationship between training set and validation set is positively influencing GS (Albrecht et al. 2011; Riedelsheimer et al. 2013; Kadam et al. 2016; Brauner et al. 2018, 2020; Herter et al. 2019). Prediction across different maize heterotic pools or highly unrelated individuals can even lead to a negative mean $\rho$ (Riedelsheimer et al. 2013; Han et al. 2018). It should be noted that the materials used for our present work were DH lines derived from two landraces both belonging to the same flint pool, but are not as closely interrelated as lines from bi-parental or interconnected families. Therefore, GS may yield higher $\rho$ for GER severity in breeding programs incorporating pre-selected lines (Albrecht et al. 2011; Brauner et al. 2018).

Across landraces, prediction accuracies close to zero were expected. Differences among landraces in the linkage phases between QTL and markers might account for this result (Brauner et al. 2018; Han et al. 2018), because GS basically utilizes the LD between SNPs and QTLs. When the TS contained only lines from PE even negative ρ was found. KE yielded somewhat higher $\rho$ than PE when used as TS, especially when the two SNPs with intermediate to major effects in KE were used as fixed effect in the GS model. This reflects the results found for each population in GWAS, i.e., the landrace having no major QTL (i.e., PE) was a poorer predictor of GER resistance in the landrace where GER QTLs could be detected (i.e., KE) while the latter was a slightly better predictor of GER resistance in PE.

In an analysis combining both landraces KE and PE for GS, the $\rho$ obtained for GER severity were reduced compared to the results obtained for individual landraces when accounting for differences in mean GER severity between populations (KE and PE) ($\rho$ =  − 0.03 for RR-BLUP and 0.36 for wRR-BLUP).

## Conclusions

This study presents phenotypic and molecular analyses of GER resistance among DH lines originating from two European maize landraces, KE and PE. The present findings suggest that favorable alleles in the two landraces can be harnessed for improving GER resistance of elite germplasm with genomic tools. Beneficial QTL alleles from KE need to be validated and then marker-assisted backcrossed (BC) into elite flint lines to increase GER resistance in this heterotic group. The BC lines should be subjected to testcrossing for selecting maturity, further adaptation traits, and finally grain yield. A subsequent selection for GER resistance on testcross basis could be beneficial, because the correlation between line and testcross performance for this resistance trait has been shown to be only moderate (Löffler et al. 2011; Martin et al. 2012c). Although no GER QTLs could be detected within PE, $\rho$ estimated by RR-BLUP was of similar magnitude than within KE, indicating that beneficial effects can be expected also from PE. In future, it might be useful to cross selected DH lines from KE and PE to accumulate their respective resistance alleles in the flint heterotic group.

## Electronic supplementary material

Below is the link to the electronic supplementary material.

Supplementary file1 (DOCX 331 kb)

Supplementary file2 (XLSX 15 kb)
