# Supplementary material for: Exploiting genetic diversity in two European maize landraces for improving Gibberella ear rot resistance using genomic tools
Source: Theor Appl Genet. 2020 Dec 3;134(3):793–805. doi: 10.1007/s00122-020-03731-9 (PMC7925457; doi:10.1007/s00122-020-03731-9)
Supplement: Supplementary file 1 — Supplementary file1 (DOCX 331 kb) [file 122_2020_3731_MOESM1_ESM.docx]

**Exploiting genetic diversity in two European maize landraces for improving Gibberella ear rot resistance using genomic tools**

David Sewordor Gaikpa, Bettina Kessel, Thomas Presterl, Milena Ouzunova, Ana L. Galiano-Carneiro, Manfred Mayer, Albrecht E. Melchinger, Chris-Carolin Schön, Thomas Miedaner

**Supplementary Tables**

**Supplementary Table 1** Repeatability values for Gibberella ear rot (GER) severity and agronomic traits in individual environments

| Trait | GON 2018 | GON 2019 | HOH 2018 | HOH 2019 |
| --- | --- | --- | --- | --- |
| **Kemater (N=250)** |  |  |  |  |
| GER (%) | 0.71 | 0.61 | 0.70 | 0.75 |
| Silking (days) | 0.93 | 0.93 | 0.95 | 0.90 |
| Plant height (cm) | 0.90 | 0.92 | 0.91 | 0.91 |
| Seedset (%) | 0.88 | 0.85 | 0.90 | 0.87 |
| **Petkuser (N=250)** |  |  |  |  |
| GER (%) | 0.81 | 0.62 | 0.76 | 0.61 |
| Silking (days) | 0.93 | 0.93 | 0.96 | 0.90 |
| Plant height (cm) | 0.88 | 0.90 | 0.91 | 0.90 |
| Seed set (%) | 0.85 | 0.82 | 0.88 | 0.80 |

GON=Gondelsheim, HOH=Hohenheim

**Supplementary Table 2** Significant SNPs detected for days to silking (DS), plant height (PHT) and seed-set (SS) and the proportion of explained genotypic variance ($p_{G}$, %) within “Kemater Landmais Gelb” population (N=236)

**See EXCEL file**

**Supplementary Table 3** Significant SNPs detected for days to silking (DS), plant height (PHT) and seed-set (SS) and the proportion of explained genotypic variance ($p_{G}$, %) within “Petkuser Ferdinant Rot” population (N=226)

**See EXCEL file**

**Supplementary Table 4** Number of candidate genes associated with ontological terminologies for Gibberella ear rot severity

**See EXCEL file**

**Supplementary Figures**


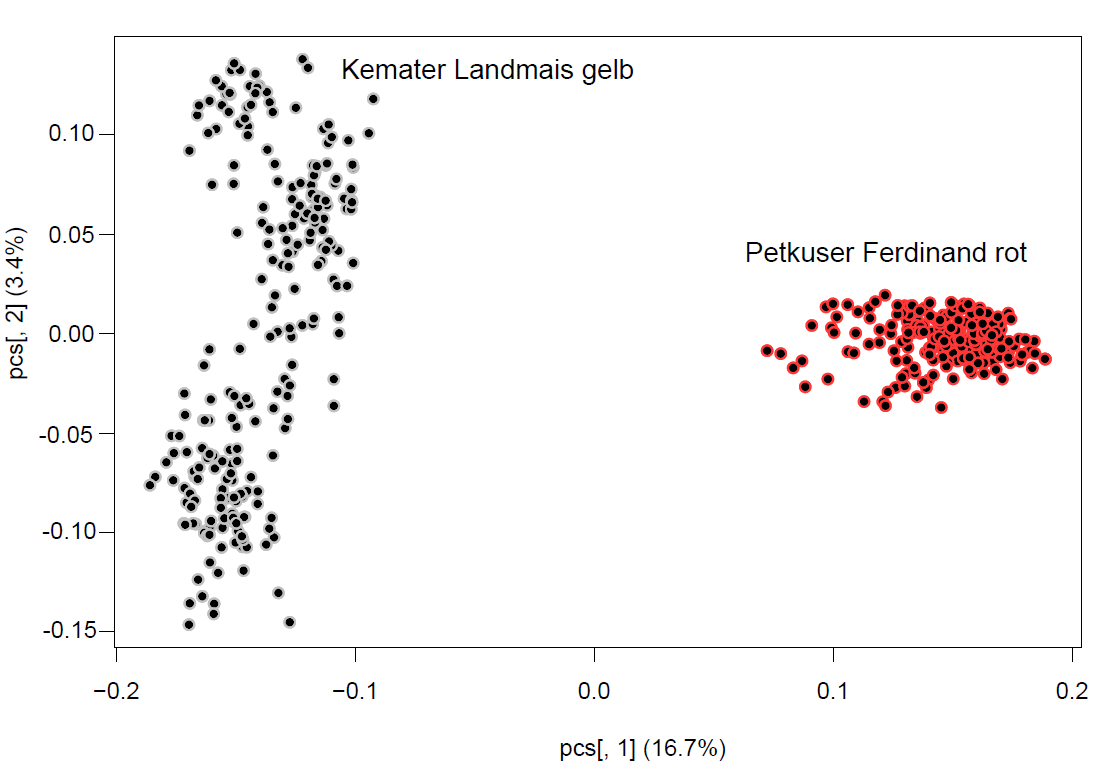


**Supplementary Figure 1** Principal component (PC) analysis of the 462 DH lines originating from two landraces based on the marker data. Percentages of variation explained by the first and second PCs are shown in the brackets


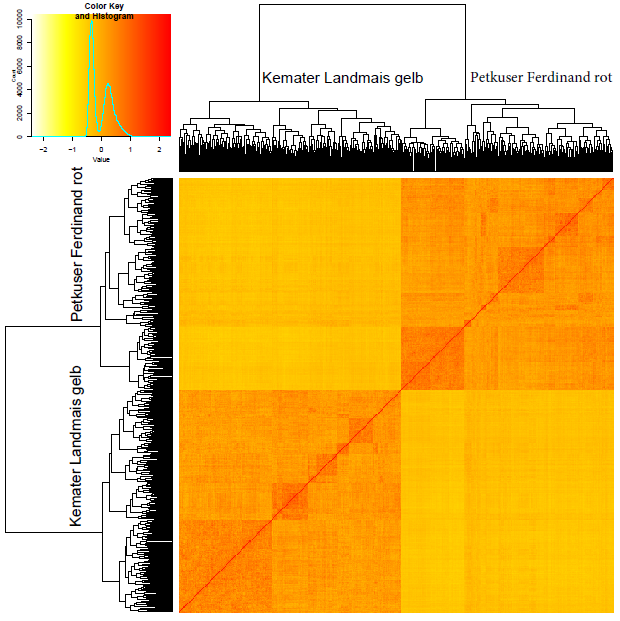


**Supplementary Figure** **2** A heat map of dendrogram and the genomic relationship matrix constructed using VanRaden algorithm in R package GAPIT.
